# The effect of childhood trauma on suicide risk: the chain mediating effects of resilience and mental distress

**DOI:** 10.1186/s12888-023-05348-w

**Published:** 2023-11-22

**Authors:** Kaimin Yao, Peiyi Chen, Hui Zhou, Jiajia Ruan, Dan Chen, Xueling Yang, You Zhou

**Affiliations:** 1 Department of Psychology, School of Public Health, Southern Medical University, Shatai South Road, Guangzhou, 510515 Guangdong China; 2 The Affiliated Brain Hospital of Guangzhou Medical University, Guangzhou, 510370 Guangdong China; 3 Student Affairs Office, Nanfang Hospital, Southern Medical University, Guangzhou, 510515 Guangdong China; 4 Department of Psychiatry, Zhujiang Hospital, Southern Medical University, Guangzhou, 510282 Guangdong China

**Keywords:** Childhood trauma, Resilience, Mental distress, Suicide risk, Chinese young adults

## Abstract

**Background:**

Suicide is the fourth leading cause of death among young people aged 15–29 years. A large number of studies have found that mental disorder in adulthood is closely related to childhood trauma, and the relationship between childhood trauma and suicide risk is influenced by resilience and mental distress. This study aimed to explore the effects of childhood trauma on suicide risk among young people and the potential roles of resilience and mental distress in the relationship between childhood trauma on suicide risk.

**Methods:**

A cross-sectional survey was conducted among 742 young adults who were in graduate school stage from multiple provinces and cities in China. The Childhood Trauma Questionnaire (CTQ-Short Form), Connor-Davison Resilience Scale (CD-RISC) and Suicide Behavior Questionnaire-Revised (SBQ-R) were used to measure young adults’ childhood trauma, resilience and suicide risk, respectively. 9-items Patient Health Questionnaire (PHQ-9) and 7-items self-report Generalized Anxiety Disorder Scale (GAD-7) were used together to measure mental distress. Correlation analysis was performed to explore the initial relationships among the main variables. Structural equation modeling (SEM) was conducted to examine the chain mediating effects of resilience and mental distress in the relationship between childhood trauma and suicide risk.

**Results:**

The structural equation modeling produced goodness of fit indices (χ^2^ /df = 3.668, *p* < 0.001, RMSEA = 0.060, NFI = 0.959, CFI = 0.969, GFI = 0.969, TLI = 0.955). Childhood trauma significantly predicted suicide risk (*β* = 0.232, *p* < 0.001) and mental distress (*β* = 0.181, *p* < 0.001), which had negative effect on resilience (*β* = -0.233, *p* < 0.001). Resilience negatively affected mental distress (*β* = -0.483, *p* < 0.001) and suicide risk (*β* = -0.142, *p* = 0.001), while mental distress positively associated with suicide risk (*β* = 0.219, *p* < 0.001).

**Conclusion:**

The current study revealed that resilience and mental distress played chain-mediating roles in the relationship between childhood trauma and suicide risk. This suggests that we should view the suicide risk of graduate students from a comprehensive perspective.

## Background

Suicide is a major public health problem worldwide, and has received wide attention in society [1]. According to the World Health Organization’s report, suicide has become the fourth leading cause of death among young adults aged 15–29 years (WHO, 2021) [2]. A meta-analysis study showed that the pooled prevalence of lifetime suicidal ideation, plans and attempts among college students were about 22.3% [95% Confidence Interval (CI): 19.5–25.3%], 6.1% (95% CI: 4.8–7.7%) and 3.2% (95% CI: 2.2–4.5%), respectively [3], indicating that suicide is pretty common to college students. Turecki and Brent’ s suicide model (2016) suggests that risk factors for suicide can be divided into population-level and individual risk factors for suicide, and the latter can be further divided into distal, developmental and proximal. According to this model, some scholar believed that childhood trauma which has a high incidence is an indispensable distal individuals risk factor [4]. Childhood trauma refers to the experiences of abuse and neglect that individuals suffered during their childhood period, mainly including various forms of emotional abuse, physical abuse, sexual abuse, emotional neglect and physical neglect [5]. Empirical studies have confirmed that childhood trauma was a significant risk factor for suicide: young adults who have experienced childhood trauma have 2 to 3 times higher suicide risk than those who have not. Severe childhood abuse was associated with a high risk of attempted suicide in adulthood [Odds Ratio (OR) = 5.18, 95% CI: 2.52–10.63] [6]. According to a report from the World Mental Health International College Student Initiative (WMH-ICS), childhood trauma is prevalent among college students worldwide with a general trauma rate ranging from 2.5% to 38.5% [7]. A meta-analysis study showed that the pooled lifetime prevalence of childhood trauma among Chinese college students was 64.7%, and the prevalence was different between regions, with the lowest prevalence of 31.3%, indicating that childhood trauma was common among Chinese college students [8]. According to previous studies, childhood trauma had a wide range of effects on individuals, including the neurobiological, behavioral, physical and psychological adverse consequences [9]. In addition, the impact of childhood trauma on individuals would continue into early adulthood, or even lifelong [10, 11]. How did childhood trauma significantly affect individuals’ suicide risk in early adulthood? Previous studies have found that resilience and mental distress were the important mediating variables between childhood trauma and suicide risk [12, 13].

Resilience refers to the ability of individuals to recover from adversity and exhibit adaptive behavior when faced with negative stress events [14, 15]. Resilience as a positive protective factor can help individuals make full use of the available resources around them and face different adversities with a positive attitude. Individuals with high levels of resilience tend to show higher cognitive flexibility and emotional regulation abilities [16], exhibit more adaptation when faced with adversity, and therefore have better ability to resist suicide risk [17]. Individuals with low levels of resilience have difficulty in accepting changes when faced with adversity and are more likely to engage in self-injurious and suicidal behavior [18]. Currently, there are different opinions on the relationship between childhood trauma and resilience: some researchers believe that childhood trauma is a risk factor for resilience [19–21]; while others believe that mild to moderate controllable childhood adversity is conducive to the development of resilience [22–24].

Mental distress as a latent variable is mainly manifested as depressive and anxious symptoms at the symptom level [25]. At present, mental distress is common among college students worldwide. Ochnik’ s study revealed that 45.9% of foreign college students had symptoms of mental distress [26]. It is reported that about 45% of Chinese college students may have mental distress symptoms [27]. Some studies have found that mental distress could positively predict college students’ suicide risk, college students who suffered from mental distress had higher suicide risk than those who did not [28, 29]. In addition, some studies have found that childhood trauma could positively predict young adults’ level of mental distress [30]. A 6-year longitudinal study found that childhood trauma was an outstanding risk factor for mental distress, and had a significantly lasting impact on mental distress [31]. Besides, there is also a close relationship between resilience and mental distress. As a self-regulation ability for successful adaptation when facing stress and adversity, good level of resilience is manifested as an effective response to environmental challenges and resistance to harmful effects of stress [32]. And the poor resilience was positively related with mental distress [33]. Scholars believed that young adults with weak resilience usually presented insufficient self-regulation ability to setbacks and stress which caused susceptibility to mental distress [34]. A longitudinal study found that young adults with weak resilience showed a significant increase in mental distress (OR = 2.94, 95% CI: 1.93–4.46) [35]. And mental distress was one of the most common proximal suicide risk factors in clinical settings [4].

Based on previous practical findings, this study aimed to examine the effects of childhood trauma on suicide risk among young people. The study proposed four hypotheses: (1) Childhood trauma significantly and positively predicts suicide risk in early adulthood; (2) Resilience acts as a mediator between childhood trauma and suicide risk; (3) Mental distress also mediates the relationship between childhood trauma and suicide risk; (4) Resilience and mental distress have a chain mediating effect between childhood trauma and suicide risk. To investigate these hypotheses, a cross-sectional study was conducted among graduate students using structural equation modeling (SEM).

## Methods

### Participants

This study was reviewed by the Ethics Committee of Southern Medical University. A cross-sectional survey was conducted among 742 young adults from multiple provinces and cities in China (mean age = 24.01, *SD* = 2.02; including 296 males and 446 females) who were in graduate school stage. We had set the following inclusion criteria and exclusion criteria: (1) could understand the meaning of questionnaires items; (2) could answer truthfully and complete all questionnaires; (3) had not been diagnosed of any forms of severe psychiatric illnesses, like bipolar disorder, schizophrenia ect.; (4) excluded cases with more than 10% missing values. And all participants were volunteered to participate in this study and signed the informed consent. For this study, we utilized the following sample size calculation formula *n* = [Z_1-α/2_^2*π(1-π)]/δ^2, which allowed for a relative error of 15%, so the absolute error δ = 0.15π, 95% confidence intervals were accepted, and Z_1-α/2_ = 1.96. According to previous research, the prevalence of mental distress among Chinese graduate students was 28%, so π = 28% [36]. The minimum sample size in this study was *n* = [1.96^2^ × 28%(1–28%)] / (0.15 × 28%)^2^ ≈ 439. Therefore, the number of participants of this study met the sample size requirements.

### Measures

#### Childhood trauma

Childhood trauma was assessed by the Chinese version of the Childhood Trauma Questionnaire-Short Form (CTQ-SF) [37, 38], a well-established tool to reflect special form and severity of childhood trauma. CTQ-SF includes five subscales (emotional abuse, physical abuse, sexual abuse, emotional neglect, and physical neglect), and each subscale have five items. Participants answered each item on a five-point Likert scale that ranges from 1 (never true) to 5 (very often true). Higher score indicates higher level of childhood trauma. The cutoff scores of five subscales among young adults are 14 for emotional neglect, 9 for physical neglect, 7 for sexual abuse, 9 for physical abuse, and 11 for emotional abuse [39]. In this study, the Cronbach's α coefficient of CTQ-SF was 0.810, and the five subscales’ coefficients of internal consistency ranged from 0.552 to 0.822.

#### Psychological resilience

Psychological resilience was measured by the Chinese version of the Connor-Davidson Resilience Scale (CD-RISC) [15, 40]. This 25-items questionnaire contains three subscales, including tenacity, strength, and optimism. Participants scored each item on a five-point Likert scale that ranges from 1 (not true at all) to 4 (true all the time). Higher score indicates higher level of psychological resilience. The Cronbach's α coefficient of CD-RISC in the present study was 0.933, and the Cronbach’s α coefficients of three subscales ranged from 0.667 to 0.878.

#### Mental distress

Consistent with previous studies [41, 42], we used the degree of depression and anxiety as indicators of mental distress in current study.

Depression was assessed by the Chinese version of 9-items Patient Health Questionnaire (PHQ-9), a valid screening tool of depressive symptoms [43, 44]. PHQ-9 consists of nine depressive symptoms, and participants were asked to report the frequency of each symptom in the past two weeks. Each item scored from 0 (not at all) to 3 (nearly every day) with total score ranging from 0 to 27. Higher total score indicates more severe depression (0–4: no depression; 5–9: mild depression; 10–14: moderate depression; 15–19: moderately severe depression; 20–27: severe depression) [45]. The Cronbach's α coefficient of PHQ-9 in the present study was 0.825.

The degree of anxiety was assessed by the Chinese version of the 7-items self-report Generalized Anxiety Disorder Scale (GAD-7) [46, 47]. GAD-7 consists of seven anxiety symptoms, and participants were needed to report the frequency of each symptom within the past two weeks. Each item scored from 0 (not at all) to 3 (nearly every day), with total scores ranging from 0 to 21. Higher total score indicates more severe anxiety (0–4: no anxiety; 5–9: mild anxiety; 10–14: moderate anxiety; 15–21: severe anxiety) [48]. The Cronbach's α coefficient of GAD-7 in the present study was 0.888.

#### Suicide risk

Suicide risk was assessed by the Chinese version of the Suicidal Behaviors Questionnaire-Revised (SBQ-R), which consists of four items, reflecting the different aspects of suicide risk [49]. The total score of SBQ-R ranges from 3 to 18. Participants whose scores reach 7 or above are identified to have clinically significant suicide risk. The Cronbach's α coefficient of SBQ-R in the present study was 0.701.

### Statistical analysis

A total of 767 questionnaires were collected through the online questionnaire, and 25 unqualified questionnaires were eliminated after screening. All statistical analyses were conducted using SPSS 22.0 and AMOS Version 7.4. First, we performed Harman’s one-factor test to examine common method bias in the current study and examined whether existed severe multicollinearity among the main variables. And we used the values of Skewness and Kurtosis to determine the distribution of the current data. Kim (2013) proposed that the data with an absolute skew value lower than 2 and an absolute kurtosis value lower than 7 could be considered as basically normal distribution [50]. Distribution of variables are slightly or significantly skewed with the Skewness ranged from 0.14 to 6.41, and the Kurtosis ranged from 0.04 to 48.58. To be specific, childhood trauma, emotional abuse, sexual abuse, physical abuse, mental distress, anxiety and suicide risk were skewed distribution. Second, those variables which met normal distribution were represented by mean (standard deviation) for descriptive analysis, and Pearson’ s correlation analysis was used for correlation analysis. And variables which were skewed distribution were represented by median (quartile) for descriptive analysis, and Spearman’ s correlation analysis was used for correlation analysis. Ming Lei & Richard (2009) analyzed the robustness of SEM analysis under different combinations of nonnormality. It shows that there are almost no differences among the different nonnormality conditions, and they proposed the usual interpretation of SEM parameter estimates can be accepted [51]. And the bootstrap in the SEM helps us to maintain the robustness of the results under nonnormal date conditions [52]. Third, we adopted Structural Equation Modeling (SEM) to examine the mediating roles of resilience and mental distress in the association between childhood trauma and suicide risk. In the current study, mental distress was indicated by two observable variables (depressive and anxious symptoms). The standards of goodness of fit indices included: ratio of Chi-square to the degree of freedom (χ2/df) should be less than 3, normed fit index (NFI) ≥ 0.90, the comparative fit index (CFI) ≥ 0.90, goodness of fit index (GFI) ≥ 0.90, the Tucker-Lewis Index (TLI) ≥ 0.90, the root mean square of approximation (RMSEA) ≤ 0.08 [53]. Bootstrap with 5000 iterations was used to calculate the 95% bias-corrected bootstrap confidence intervals of direct and indirect effects.

## Results

### Tests for common method bias and multicollinearity

The results of Harman’s one-factor test indicated that the first common factor explained 17.98% of the total variance, which was far below 40% [54]. Taking suicide risk as dependent variable, and childhood trauma, resilience, and mental distress as independent variables, the results of collinearity test showed that the variance inflation factors (VIFs) ranged from 1.115 to 1.334, which were close to 1 [55]. We could consider that no severe common method bias and multicollinearity exist in our data.

### Descriptive statistics and correlation analysis

Detailed descriptions of sociodemographic characteristic of the current sample were presented in Table 1. 37.7% of participants (*n* = 280) reported at least one type of trauma. About 24.1% of participants had depression, and 19.6% of participants had anxiety. 7.8% of participants exceeded the cut-off for suicide risk (See Table 1). Correlations among the main variables were summarized in Table 2. Childhood trauma was negatively correlated with resilience (*r* = -0.358, *p* < 0.001), and positively correlated with depression, anxiety, and suicide risk (*r* = 0.262, *p* < 0.001; *r* = 0.266, *p* < 0.001;* r* = 0.276, *p* < 0.001). And emotional abuse, emotional neglect, sexual abuse, physical abuse, and physical neglect were all positively correlated with suicide risk (*r* = 0.257, *p* < 0.001; *r* = 0.194, *p* < 0.001;* r* = 0.106, *p* = 0.004; *r* = 0.132, *p* < 0.001; *r* = 0.159, *p* < 0.001). Resilience was negatively associated with depression, anxiety, and suicide risk (*r* = -0.451, *p* < 0.001; *r* = -0.427, *p* < 0.001;* r* = -0.289, *p* < 0.001). Both depression and anxiety were positively correlated with suicide risk (*r* = 0.290, *p* < 0.001; *r* = 0.312, *p* < 0.001).

**Table 1:** Socio-demographic characteristics of the sample

Variables	Description	Number (%)
**Age**	24.01 ± 2.02	
**Gender**		
Male		296 (39.9)
Female		446 (60.1)
**Childhood trauma**	32 (29, 37)	
Emotional Abuse	6 (5, 7)	22 (3.0)
Emotional Neglect	8.98 ± 3.38	71 (9.6)
Sexual Abuse	5 (5, 5)	41 (5.5)
Physical Abuse	5 (5, 5)	16 (2.2)
Physical Neglect	7.67 ± 2.46	231 (31.1)
At least have one trauma		280 (37.7)
**Depression**	2.81 ± 2.92	
No		563 (75.9)
Mild		164 (22.1)
Moderate		11 (1.5)
Moderately severe		2 (0.3)
Severe		2 (0.3)
**Anxiety**	1 (0, 4)	
No		595 (80.4)
Mild		130 (17.6)
Moderate		12 (1.6)
Severe		3 (0.4)
**Suicide risk**	3 (3, 5)	
No		684 (92.2)
Yes		58 (7.8)

*Note*: description, Mean ± SD or median (quartile)

**Table 2:** Descriptive analysis and correlation analysis among the main variables (*n* = 742)

	**Description**	1	2	EA	EN	SA	PA	PN	3	4	Depression	Anxiety	5
1. Age	24.01 ± 2.02	-											
2. Childhood trauma	32 (29, 37)	0.016	-										
EA	6 (5, 7)	0.005	0.518^***^	-									
EN	8.98 ± 3.38	0.056	0.857^***^	0.260^***^	-								
SA	5 (5, 5)	-0.037	0.289^***^	0.172^***^	0.160^***^	-							
PA	5 (5, 5)	0.042	0.366^***^	0.316^***^	0.234^***^	0.239^***^	-						
PN	7.67 ± 2.46	0.012	0.766^***^	0.256^***^	0.529^***^	0.130^***^	0.169^***^	-					
3. Resilience	67.54 ± 13.43	0.066	-0.358^***^	-0.191^***^	-0.303^***^	-0.105^***^	-0.097^**^	-0.252^***^	-				
4. Mental distress	4 (1, 8)	0.039	0.294^***^	0.258^***^	0.201^***^	0.121^***^	0.129^***^	0.200^***^	-0.481^***^	-			
Depression	2.81 ± 2.92	0.037	0.262^***^	0.235^***^	0.181^***^	0.120^***^	0.131^***^	0.198^***^	-0.451^***^	0.929^***^	-		
Anxiety	1 (0, 4)	0.052	0.266^***^	0.228^***^	0.181^***^	0.094^*^	0.090^*^	0.190^***^	-0.427^***^	0.902^***^	0.695^***^	-	
5. Suicide risk	3 (3, 5)	-0.085^*^	0.276^***^	0.257^***^	0.194^***^	0.106^**^	0.132^***^	0.159^***^	-0.289^***^	0.323^***^	0.290^***^	0.312^***^	-

*EA* Emotional Abuse, *EN* Emotional Neglect, *SA* Sexual Abuse, *PA* Physical Abuse, *PN* Physical Neglect

*Note*: significance was set as ^*^ *p* < 0.05; ^**^ *p* < 0.01; ^***^ *p* < 0.001; description, Mean ± SD or median (quartile)

### Mediation analyses

**Fig. 1:**
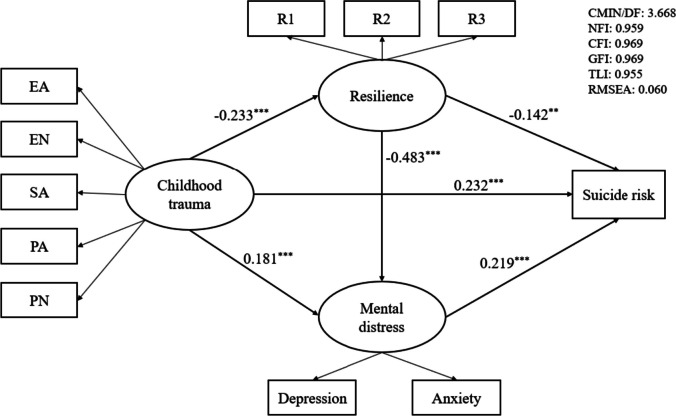
Resilience and mental distress as mediators in the relationship between childhood trauma and suicide risk. Note: significance was set as ^*^ *p* < 0.05; ^**^ *p* < 0.01; ^***^ *p* < 0.001; EA, Emotional Abuse; EN, Emotional Neglect; SA, Sexual Abuse; PA, Physical Abuse; PN, Physical Neglect; R1, tenacity; R2, strength; R3, optimism; CMIN/DF, ratio of Chi-square to the degree of freedom; NFI, Normed Fit Index; CFI, Comparative Fit Index; GFI, Goodness of Fit Index; TLI, Tucker-Lewis Index; RMSEA, the root mean square of approximation; CMIN/DF: 3.668; NFI: 0.959; CFI: 0.969; GFI: 0.969; TLI: 0.955; RMSEA: 0.060

Before conducting the mediation analyses, we test the relationship between childhood trauma and suicide risk. The results showed that childhood trauma significantly predicted suicide risk (*β* = 0.295, *p* < 0.001). Then, we carried out a test of the mediation model. This model produced acceptable fit indices (χ^2^ /df = 3.668, *p* < 0.001, RMSEA = 0.060, NFI = 0.959, CFI = 0.969, GFI = 0.969, TLI = 0.955). All the path coefficients were significant in this model (See Fig. 1). Specifically, childhood trauma had negative effect on resilience (*β* = -0.233, *p* < 0.001), and then resilience negatively affected suicide risk (*β* = -0.142, *p* = 0.001). Besides, childhood trauma could also predict higher mental distress (*β* = 0.181, *p* < 0.001), and then mental distress positively associated with suicide risk (*β* = 0.219, *p* < 0.001). Moreover, the current results also revealed that the chain mediating effects of resilience and mental distress in the relationship between childhood trauma and suicide risk were significant, and the resilience could negatively predict mental distress (*β* = -0.483, *p* < 0.001). The analyses also found that childhood trauma had significant direct effect on suicide risk in this model (*β* = 0.232, *p* < 0.001). Detailed effect sizes of direct and indirect paths were presented in Table 3.

**Table 3:** The bootstrap analysis of paths and effects (*n* = 742)

Effect	Path	Effect size			
		**Effect**	**Ratio of effect**	**SE**	**95% CI**
Direct effect	childhood trauma → suicide risk	0.232	70.30%	0.041	(0.149, 0.312)
Indirect effect	childhood trauma → resilience → suicide risk	0.033	10.00%	0.012	(0.014, 0.061)
	childhood trauma → mental distress → suicide risk	0.040	12.12%	0.012	(0.020, 0.069)
	childhood trauma → resilience → mental distress → suicide risk	0.025	7.58%	0.007	(0.013, 0.042)
Total effect		0.330		0.040	(0.250, 0.408)

*Note*: *SE* Standard Error, *95% CI* 95% bias-corrected Confidence Interval

## Discussion

Recently, the frequent suicide cases among graduate students have drawn wide attention. Our study found that 7.8% of graduate students had suicide risk, and 37.7% of graduate students reported had experienced at least one type of trauma. Physical neglect was the most common trauma (31.1%), followed by emotional neglect (9.6%), sexual abuse (5.5%), emotional abuse (3.0%) and physical abuse (2.2%), which suggests that childhood trauma, especially neglect, was prevalent among graduate students. The correlation analysis found that different forms of childhood trauma were differently correlated with suicide risk among Chinese graduate students, and especially we found emotional abuse and emotional neglect were more closely related to suicide risk. It suggested that we should pay more attention to the impact of childhood trauma on suicide risk among Chinese graduate students, especially emotional trauma.

We also found that childhood trauma was positively correlated with mental distress and suicide risk, which was consistent with the previous research [56–58], indicating that the more severe childhood trauma, the more likely individuals developed mental distress and suicide risk in early adulthood. Previous studies suggested that childhood trauma was also an important factor for resilience [19–21], and the current finding further confirmed that there was a significant negative correlation between childhood trauma and resilience. And the SEM proved the hypothesis that resilience and mental distress mediated the relationship between childhood trauma and suicide risk. The current results showed that graduate students’ childhood trauma not only directly affects suicide risk, but also indirectly affects it through the mediation of resilience and mental distress. The mediation is achieved through the following three paths: (1) the independent mediating effect of resilience; (2) the independent mediating effect of mental distress; (3) the chain mediating effect of resilience and mental distress.

The present findings showed that childhood trauma could affect graduate students’ suicide risk through the mediating effect of resilience, which accounted for 10% of the total effect. Our findings suggest that childhood trauma may hinder the development of resilience, and low levels of resilience may mediate the suicide risk associated with childhood trauma. Previous studies have observed that childhood trauma, as a distal risk factor, can lead to structural and functional abnormalities in specific brain regions such as the corpus callosum, hippocampus, and amygdala, which are closely related to resilience [59–61]. In the case of graduate students with weak resilience, they may struggle to self-regulate when confronted with negative life events, making them more prone to emotional instability and engaging in self-injurious or suicidal behaviors. Considering the dynamic nature of resilience, it is important to note that individual protective factors and external support from family, school, society, and peer groups can enhance an individual's resilience [62]. Consequently, resilience becomes a promising target for preventing and reducing suicide risk among graduate students who have encountered childhood trauma [17]. Besides, this study also found that mental distress played an independent mediating role between childhood trauma and suicide risk, accounting for 12.12% of the total effect. The more childhood trauma they had experienced, the higher their mental distress levels might have, which in turn greatly increase their suicide risk. Some neuroimaging studies supported the current findings: some key brain regions related to mental distress were dampened in traumatic individuals such as the prefrontal cortex, anterior cingulate cortex, amygdala and hippocampus [63]. What’s more, childhood trauma could also lead to Hypothalamic–Pituitary–Adrenal axis (HPA axis) dysregulation and an unbalance in serotonin and dopamine which could cause more mental distress, and finally increase suicide risk [64, 65]. McLaughlin's study highlighted that childhood trauma can heighten an individual's perception of negative events or stress, especially in those with mental disorders, thus making them more vulnerable to developing emotional symptoms [66]. Moreover, childhood trauma can also lead to difficulties in emotional regulation and increased emotional reactivity towards negative stimuli [59]. Another study indicated that individuals who have experienced childhood trauma are more likely to perceive these events as challenging to cope with or uncontrollable, resulting in heightened emotional responses [67]. In summary, individuals with traumatic experiences demonstrate a higher sensitivity to perceive stress, increased arousal and reactivity of emotions, and poorer abilities to regulate and cope with negative emotions. This heightened susceptibility to mental distress ultimately contributes to an increased risk of suicide.

Moreover, the current study found that resilience and mental distress played significant chain mediating roles between childhood trauma and suicide risk, showing that childhood trauma could affect graduate students’ mental distress level through resilience, and then eventually affected suicide risk. Resilience could negatively predict graduate students’ mental distress level, which means graduate students with great resilience are more capable of coping effectively with setbacks when facing adversity, thus avoiding adverse emotional reactions or alleviating mental distress. Previous studies have consistently found strong interactions between childhood trauma and resilience, indicating that individuals with high childhood trauma and low resilience are more likely to experience higher levels of mental distress [68]. In our study, we specifically considered childhood trauma as a significant distal risk factor for suicide. Our findings suggest that the mechanism through which childhood trauma increases suicide risk may involve the dampening of individuals' resilience, which can be seen as a trait, subsequently impacting their state of mental distress. Resilience and mental distress can be viewed as trait and state factors, respectively. The chain mediating effect of weakened resilience and heightened mental distress may substantially increase the suicide risk among young adults who have experienced childhood trauma, providing a more comprehensive explanation for the occurrence and progression of suicide. It suggested that we should view the suicide risk of graduate students from a more comprehensive perspective. The study provided a new perspective and some empirical basis for exploring the effective prevention and intervention of suicide risk among graduate students with childhood trauma.

### Limitations

Despite the benefits of this study for further understanding of the potential role of resilience and mental distress in the relationship between childhood trauma and suicide risk, there are still some limitations. First, this study used self-report scales, which could hardly avoid reporting bias due to social desirability, response set, and subconscious motivation. Secondly, it might recall bias of childhood trauma among the participants surveyed in the study. Third, the current study only recruited graduate students in China only. Future studies could examine and supplement current results in multi-populations or cross-culture studies around the world. Finally, this study was a cross-sectional study that could not draw clear causal conclusions. Future studies should be conducted from longitudinal and experimental design on the associations among childhood trauma, suicide risk, resilience and mental distress.

## Conclusions

The present study found that resilience and mental distress played chain-mediating roles in the relationship between childhood trauma and suicide risk. These findings emphasize the significance of resilience and mental distress in preventing suicide risk and highlight the importance of considering a more comprehensive perspective when assessing the suicide risk among graduate students.

## Data Availability

The datasets generated and analyzed during the current study are available from the corresponding author on reasonable request.

## Abbreviations

CTQ-SF: The Childhood Trauma Questionnaire-Short Form
CD-RISC: Connor-Davison Resilience Scale
SBQ-R: Suicide Behavior Questionnaire-Revised
PHQ-9: 9-Items Patient Health Questionnaire
GAD-7: 7-Items self-report Generalized Anxiety Disorder Scale
SEM: Structural Equation Modeling
WHO: The World Health Organization
WMH-ICS: The World Mental Health International College Student Initiative
SD: Standard Deviation
SE: Standard Error
95% CI: 95% Bias-corrected Confidence Interval
EA: Emotional Abuse
EN: Emotional Neglect
SA: Sexual Abuse
PA: Physical Abuse
PN: Physical Neglect
R1: Tenacity
R2: Strength
R3: Optimism
CMIN/DF: Ratio of Chi-square to the degree of freedom
NFI: Normed Fit Index
CFI: Comparative Fit Index
GFI: Goodness of Fit Index
TLI: Tucker-Lewis Index
RMSEA: The root mean square of approximation
HPA axis: Hypothalamic-Pituitary-Adrenal axis
